# A qualitative study of chronic obstructive pulmonary disease patient perceptions of the barriers and facilitators to adopting digital health technology

**DOI:** 10.1177/2055207619871729

**Published:** 2019-08-25

**Authors:** Patrick Slevin, Threase Kessie, John Cullen, Marcus W. Butler, Seamas C Donnelly, Brian Caulfield

**Affiliations:** 1 The Insight Centre for Data Analytics, University College Dublin, Ireland; 2 University College Dublin, Ireland; 3 Tallaght University Hospital, Dublin, Ireland; 4 St. Vincent’s University Hospital, Dublin, Ireland; 5 Trinity College Dublin, Dublin, Ireland

**Keywords:** COPD, digital health, qualitative, barriers, facilitators

## Abstract

**Objective:**

Non-adherence to self-management plans in chronic obstructive pulmonary disease (COPD) results in poorer outcomes for patients. Digital health technology (DHT) promises to support self-management by enhancing the sense of control patients possess over their disease. COPD digital health studies have yet to show significant evidence of improved outcomes for patients, with many user-adoption issues still present in the literature. To help better address the adoption needs of COPD patients, this paper explores their perceived barriers and facilitators to the adoption of DHT.

**Methods:**

A sample of convenience was chosen and patients (*n* = 30) were recruited from two Dublin university hospitals. Each patient completed a qualitative semi-structured interview. Thematic analysis of the data was performed using NVivo 12 software.

**Results:**

Barrier sub-themes included lack of perceived usefulness, digital literacy, illness perception, and social context; facilitator sub-themes included existing digital self-efficacy, personalised education, and community-based support.

**Conclusion:**

The findings represent a set of key considerations for researchers and clinicians to inform the design of patient-centred study protocols that aim to account for the needs and preferences of patients in the development of implementation and adoption strategies for DHT in COPD.

## Introduction

Chronic obstructive pulmonary disease (COPD) is a manageable, largely preventable respiratory disease and is the fourth leading cause of death globally.^1^ In the European Union, annual costs of COPD have been estimated at €23.3 billion with expenditure primarily attributed to exacerbation-related hospitalisations.^2^ Exacerbations that require hospitalisation are related to greater mortality and morbidity compared with those treated in out-patient settings.^3^ Early recognition of an exacerbation and timely intervention can reduce the risk of hospitalisation but achieving this requires effective management of the disease in the community.^4^ It is well-established that patients are now expected to take an active role in the management of their disease.^5^ Engaging in pro-active self-management in COPD is linked to enhanced health-related quality of life, reduced admissions and decreased duration of exacerbations.^6–8^ However, self-management does not appear to work consistently in COPD, with non-adherence to therapeutic regimes and action plans frequently attributed to poorer outcomes.^9–11^

Digital health technology (DHT), including self-monitoring devices (e.g. oximeters and pedometers) and healthcare ‘apps’, has been identified as an innovative model to help optimise the provision of COPD care by supporting patients to enhance the knowledge and sense of control they possess over self-management practices such as self-monitoring and problem solving.^12–14^ Healthcare professionals (HCPs) can benefit from longitudinal datasets captured outside the clinic to inform decision-making and support self-management through the personalisation of treatment plans that are more aligned with patient needs and preferences.^15–18^

The promises are appealing, however digital health research in COPD has yet to have a significant impact on routine care, with convincing evidence of improved outcomes for patient self-management in limited supply.^19–21^ A common problem facing COPD digital health studies is cultivating user adoption, with low adherence and sustained engagement levels with deployed interventions frequently cited for both patients and HCPs.^22,23^ For patients, user-experience issues have been found to negatively impact on adoption such as digital and health literacy,^24,25^ the usability of the technology^26,27^ and the burden of completing added self-management tasks using technology.^28^

Such issues are generally identified through post-study user-evaluations, with adoption needs rarely addressed or prioritised in the design or implementation phases of digital health studies in COPD.^29^ Furthermore, previous research has highlighted the tendency for digital health studies to define predetermined research goals, which often lead to the development of ‘one-size-fits-all’ solutions that prioritise clinical outcomes at the expense of accounting for individual needs.^30,31^

This raises questions about the value of identifying the adoption needs of COPD patients to inform the design and implementation of a DHT. As Clemensen et al. suggest, understanding user needs prior to the design of a digital health intervention can help researchers establish patient issues before the specifications of a solution are considered.^32^ Indeed, recent systematic reviews have concluded that further qualitative research investigating the user needs of COPD patients is required to highlight the ‘key ingredients’ that will better inform the development of implementation and adoption strategies for digital health interventions in a patient-centred manner.^19,33^ Although current COPD research in mHealth has begun focusing on patient needs in the early-development phase,^34^ literature in this field is sparse.

It will be useful therefore to explore the adoption needs of COPD patients, particularly to investigate the potential barriers and facilitators they perceive to the use of DHT. As such, this study employed a qualitative design to explore the following research question: what are COPD patients’ perceived barriers and facilitators to using DHT? The aim was for the findings of this study to contribute to patient-centred design considerations to support researchers and clinicians in the development of implementation and adoption strategies to mitigate adoption issues in COPD digital health interventions.

## Methods

### Study design

This research employed a qualitative study design using semi-structured interviewing.

### Recruitment and sample

Patient recruitment took place in the respiratory clinics of two university hospitals. A sample of convenience was chosen for pragmatic reasons. Patients were identified by respiratory consultants (MB and JC) and potential participants were then invited to partake in the study. Exclusion criteria were any existing cognitive or psychotic disorders, or severe life-limiting co-morbidities, such as lung cancer. Inclusion criteria included a confirmed diagnosis of COPD guided by the GOLD guidelines.^35^ An information leaflet and consent form were given to interested patients and a 48-h reflection period was provided prior to the researcher (PS) contacting the patient to confirm participation. Upon confirmation, a date and time convenient to the patient was scheduled for the interview. The number of patients that declined participation was not gathered. Interviews were conducted at the patients’ homes and written consent was obtained before each interview.

### Procedure

Semi-structured interviews were conducted by PS who is an experienced qualitative researcher. An interview topic guide (Table 1) was used and patient’s perceptions of the barriers and facilitators to adopting DHT were explored. The combination of semi-structured interviewing and open-ended questions allowed for new topics of conversation to emerge and these were explored with the patients.^36^

**Table 1.** Interview Topic Guide.

Topic	Questions
Demographics	Age, marital status, occupation status, highest education attained; technology (mobile or smartphone, PC, laptop), smoking history.
Disease experience	Can you discuss your experience of your COPD?What is the role of family and friends when managing your COPD? What types of self-management practices do you perform? How do you feel about self-managing? Can you discuss how you manage your symptoms? Can you tell me about an exacerbation you had? Can you discuss the last time you ended up in the general practitioner (GP) clinic and/or hospital?
Healthcare experience	Can you tell me about the kinds of care you receive or have received for your COPD? How do you feel about the care you receive for your COPD? Is the care you are receiving meeting your needs?
Health data and DHT	Do you record/log information about your health? If so, why/how? If not, why? Do you think you could provide your HCP (e.g. GP or consultant) with more information about your health day-to-day? What types of information do you think your doctor should have about your health? How would you feel about using a digital health technology e.g. oximeter, COPD-related smartphone app, spirometer, self-reported outcomes platform etc., to generate health information/data about yourself? What do you think about capturing information/data in the home? How might collecting health information/data at home impact on how you manage your COPD? Can you discuss why you might share information/data you collect with your HCP? How do you think these types of information/data could be used by your HCP to manage and treat your COPD? Can you tell me how these types of data could be collected that would be suitable for you and your needs?

### Data analysis

Interviews lasted between 60 and 90 min. They were audio-recorded with a dictaphone, transcribed verbatim and anonymised. NVivo 12 software was used to perform thematic analysis of the data (QSR International Pty Ltd, Victoria, Australia). Thematic analysis of the transcripts was conducted in line with Braun and Clarke^37^ and the topic guide provided an initial structure for developing the codebook.^38^ A subset of transcriptions were initially analysed by PS and TK to iterate and finalise the codebook.^39,40^ Analysis involved reading each transcript closely, identifying emergent patterns, labelling codes to data, and generating themes and sub-themes.^37^ Analytical rigour was ensured by PS and TK coding the data independently and afterwards scrutinising, comparing and discussing the coding to resolve any discrepancies identified.^41^ Analysis was conducted after every 10 interviews and data saturation was determined at 30 participants when no new patterns or themes were emerging from the analysis.^42^

## Results

In total, 30 interviews were completed. Sample characteristics can be observed in Table 2. Of the 30 participants, only two had experience of using DHT, both were using an oximeter. The following themes and sub-themes were identified in the data: 1) barriers to adopting DHT, with three sub-themes: lack of perceived usefulness, digital literacy, and illness perception and social context; and 2) facilitators to adopting DHT, including three sub-themes: existing digital self-efficacy, personalised education, and community-based support. Participant's age, disease classification and highest educational level attained accompany the quotes presented.

**Table 2.** Sample Characteristics*.

Characteristics	Data
Gender and age	
No. male/female	17/13
Age range	46–88 yrs
Smoking history	
Current smoker	5
Ex-smoker	25
Occupational status	
Homemaker	1
Carer’s allowance recipient	1
Retired	20
Employed	5
Unemployed	3
Marital status	
Married	19
Widowed	7
Single	3
Separated	1
Technology	
Smartphone	16/30
Laptop	18/30
Both smartphone and laptop	15/30
No smartphone, laptop or PC	11/30
Oximeter	2/30
Highest education level attained	
Primary	12
Secondary	6
Third level or above	12
COPD severity classification**	
Mild (GOLD Stage 1: FEV_1_ ≥ 80% predicted)	2
Moderate (GOLD Stage 2: 50% ≤ FEV 1 <80% predicted)	16
Severe (GOLD Stage 3: 30% ≤ FEV 1 <50% predicted)	9
Very severe (GOLD Stage 4: FEV1 <30% predicted)	3

*Sample characteristics data self-reported at interview.

** Data collected from patient medical charts.

### Barriers to adopting DHT

#### Lack of perceived usefulness

There was a lack of perceived usefulness highlighted on several fronts by patients. It was felt by patients that their current self-management practices were already overwhelming and time-consuming, they were therefore not receptive to the addition of a DHT.

I spend enough time taking medications and inhalers, so if I have to start using a new gadget, I'm not sure there's a place for it, as I said, it gets overwhelming (aged 70, Stage 2, secondary).

Patients also discussed their preference for clinical visits with concerns being raised about the consequences for clinical decision-making in situations where digitally shared health information replaces traditional face-to-face conversations.

A conversation is worth so much to me, it is hard enough to get time with the GP, let alone the doctor in the hospital. So, when you get them you want to tell them everything that is happening. But if you are telling them how you are with something they have to read, are they going to really get what they need? (Aged 64, Stage 2, undergraduate).

Others perceived little benefit accruing from HCPs having continuous access to captured data, for example, patients felt that the continuous sharing of health data would not disrupt current healthcare practices such as visiting the clinic.

If the oximeter levels were down low I’m not sure what he [General Practitioner, GP] could do if he had that information in the meantime, I’d still end up going in which I would have done anyways (aged 82, Stage 2, apprenticeship).

#### Digital literacy

Digital literacy was highlighted as an adoption barrier to the use of DHT. Patients’ responses related to their sense of technological self-efficacy and how this could negatively affect their ability to correctly perform the required tasks appropriately.

It’s a confidence thing isn’t it? I couldn’t do it by myself. I’d just be worried I’d do it and it wasn’t right then it might waste the doctor’s time (aged 65, Stage 3, primary).

It was also highlighted that if patients do not possess particular digital literacy skills this would create a barrier to their ability to interpret and act upon device readings in a beneficial manner.

If I do not understand what the readings mean for me, then I can’t really do anything about it, I am just seeing a number and that’s useless (aged 67, Stage 4, undergraduate).

#### Illness perception and social context

Illness perception emerged as a barrier to patients’ readiness to adopt DHT, for instance, patients may perceive that their current physical functioning does not align with the goals of the prescribed digital intervention.

I am getting to a stage where I am not well enough to try anything new because say something like the exercise apps, they apply to someone who is able to go out and walk. I’m really not there at the moment (aged 73, Stage 3, apprenticeship)

Patients also discussed the impact of their social context as an adoption barrier. The burdens associated with living on one’s own were emphasised such as the lack of familial support to help with managing their COPD, and this was extended to the adoption of digital interventions.

Like I’m totally dependent on myself, I’ve no wife or children. It puts a lot of burden on me to deal with all the appointments and going to my GP … so I think I would be at a disadvantage when taking this type of technology on (aged 69, Stage 2, Primary).

### Facilitators to adopting DHT

#### Existing digital self-efficacy

Although digital literacy and technological self-efficacy were perceived as a barrier by many, other patients felt that because they have established digital skills and knowledge from using various digital technologies this would facilitate easier adoption.

Because I am already using a smartphone I would be more open to trying things like recording information at home. The technology to do that wouldn't be as big a problem for me either, so I wouldn't need much training (aged 46, Stage 2, Undergraduate).

I don’t go an hour of the day without checking my phone and I see tracking as part of life now. I have GPS on my phone and that tells me everywhere I’ve been and how to get places, so I am happy to take that into my healthcare (aged 61, Stage 2, Undergraduate).

#### Personalised education

The primary adoption facilitator patients discussed was education. However, it was perceived by patients that a one-size-fits-all education approach would not be appropriate for DHT. For example, many felt that the education received should be personalised and should reflect the clinical and psychosocial factors of an individual’s disease.

The education you are getting shouldn’t be general, it should take your illness and quality of life into the equation … because if it’s just general that might not give you the best results (aged 65, Stage 2, secondary).

Patients also highlighted the need to provide this education as early as possible to help demystify the use of digital interventions in the management of their disease.

Teaching people as early as they can with technology would take the mystery out of it and might mean that people wouldn’t be as afraid of it (aged 57, Stage 4, secondary).

#### Community-based support

Patients spoke about their desire for community-based support to ease the adoption process. Many patients mentioned a preference for receiving ongoing education and supervision as their digital competencies develop.

It would be great to have the nurse come out here [to their home] to show you and to do rounds until the person can cope on their own with it (aged 74, Stage 4, primary).

Patients also highlighted their preference for a social learning environment to support adoption.

I’d see it working best if you were able to do it in a group, like if a group of 6 people were given a device or an app on their phone and were adding information together … you could see how everyone else is doing along with you and learn from each other and maybe talk about your information with them too (aged 69, Stage 2, primary).

## Discussion

This study identified new perspectives regarding the barriers and facilitators perceived by COPD patients for the adoption of DHT. The results identified three primary barriers. It is well-established that perceived usefulness is a core determinant of a person’s intention to adopt a technology.^43,44^ For self-management technology, perceived usefulness refers to the degree to which a person believes the technology could improve or enhance their ability to manage the disease.^45^ However, the perceived usefulness of DHT was questioned by participants in this study. Patients expressed that due to the burden associated with their existing self-management task-load,^46,47^ the addition of a DHT was not perceived as appropriate. Previous research in COPD posits that the self-management benefits associated with DHT may not be perceived by all patients because of the required commitment to actively engage in long-term management; however they suggest that this barrier can be addressed by assessing and accounting for the patients’ particular self-management approaches during implementation.^48^

Some patients were not receptive to the prospect of digital health data replacing the opportunity to share information through face-to-face conversations with HCPs, with concerns raised about the capacity of digital data to adequately inform treatment decision-making. These concerns align with recent findings regarding the use of digital health data by HCPs, who often perceive these data to offer inadequate evidence, or experience a lack of confidence when interpreting and actioning treatment decisions.^49,50^ Other patients were unconvinced about the effectiveness of their HCPs having continuous access to health data generated in the home, for example, patients did not perceive this would reduce the need for clinical visits. Interestingly, reducing clinical visits has long been an aim of COPD digital health studies, yet very few have achieved significant outcomes in this area.^51–53^ This raises questions about the level of priority patients’ needs are afforded in the development of study aims for digital health research in COPD.

Digital literacy was widely discussed as an adoption barrier to DHT. Digital literacy refers to

interplay of individual and social factors in the use of digital technologies to search, acquire, comprehend, appraise, communicate and apply health information in all contexts of health care with the goal of maintaining or improving the quality of life throughout the lifespan’.^54^

Aspects of this definition were found in patients’ perceptions of digital literacy in this study. With regard to individual and social factors, patients drew attention to the negative impact a reduced sense of technological self-efficacy can have on a person’s perceived ability to use a DHT appropriately. This perception may be explained by the mean age of this study sample at 68.2 ± 10.1, who traditionally, as an over-65 cohort, has lower computer literacy and technological self-efficacy levels.^55,56^ However, this is consistent with the age-profiles observed in COPD populations,^57^ therefore, because age has been found to negatively correlate with technological self-efficacy,^58^ addressing the digital literacy needs of patients participating in COPD digital health studies should help to ease such age-related adoption issues.

Patients also perceived potential barriers arising from their ability to comprehend the data generated by DHT. This was articulated in the findings as uneasiness about how to action the data provided to make a health-related decision. The potential of DHT to create positive patient outcomes relies heavily on the individual possessing a unique set of digital literacy skills to properly interpret and apply the data to their health. However, the impact of digital literacy on the adoption of DHT is an under-researched topic, even though it is recognised as a road-block to reducing the digital divide.^59^ Participatory design approaches are recommended in the development of digital health interventions to ensure that the spectrum of health and digital literacy needs in patient populations are catered for.^60^

The findings also revealed that a patient’s illness perception and social context are perceived as barriers to the adoption of DHT. Illness perception refers to the ideas, views and beliefs that a patient has about their symptoms and illness.^61^ The impact of illness perception on DHT in COPD has not received adequate attention, but this research has shown that if a patient does not believe their current health status is conducive to the proposed digital intervention, this can create an adoption barrier. Additionally, patients perceived that their social context would be a factor that affected the adoption of DHT. This was particularly pertinent for those patients who live on their own, or for those lacking a strong social ecology consisting of friends and family that could otherwise support them to manage the use and adoption of DHT. Previous research has shown that the presence or perception of a strong social support structure improves patient compliance to self-management plans in COPD and across chronic disease in general,^62,63^ yet family support is under-studied with respect to adopting DHT.

Three facilitators to aid adoption of DHT were identified in the findings. Although digital literacy was a perceived barrier for the majority, there were patients who felt that the adoption of DHT would be eased due to their existing knowledge and skills with digital technology such as smartphones. Prior knowledge and experience of technology have been shown to increase a person’s intention to use, as they facilitate understanding of the technologies’ purpose, while helping to foster ease of use through intuitive interaction.^44,64^ Patients felt that adoption can be facilitated through the provision of personalised DHT education that takes into consideration the individual needs and preferences of the patient. It was also felt that DHT education should be provided to COPD patients as early as possible to help demystify technology and mitigate adoption barriers caused by unfamiliarity. To facilitate smoother user adoption, patient-centred approaches for delivering technology education have been proposed for eHealth implementation strategies,^65–67^ while researchers conducting a recent wearable and mHealth study in COPD found that their educational component should have been tailored to the individual sedentary behaviours of patients to better support adherence.^68^

The findings also showed a preference from patients for the DHT adoption process to involve a variety of community-based supports. Patients referenced the desire for ongoing supervision from HCPs as their digital competencies evolve. Although ongoing support may be outside the resource capabilities of many HCPs, patient–clinician partnerships have been emphasised to facilitate adoption, as they afford HCPs the opportunity to work collaboratively with patients to aid with the development of data synthesising and decision-making skills.^69^ Others perceived adoption could be facilitated by the creation of a peer-to-peer social learning environment. Peer coaching has shown success in mHealth research aiming to increase the physical activity of individuals with Parkinson’s; they benefitted from cooperative goal-setting and regular feedback.^70^

## Limitations

This study used qualitative methods to gain an in-depth understanding of the barriers and facilitators COPD patients perceived to adopting DHT. The findings were strengthened by the rigour demonstrated in data collection and the use of NVivo 12 software to aid analysis. However, when considering the generalizability of findings, the relatively small sample size should be viewed as a limitation. For instance, the mean age of this cohort is 68.2 ± 10.1 with 11/30 patients having no smartphone, laptop or PC; this may explain why this cohort placed an emphasis on digital literacy as a barrier and the need for technology-focused education as a central aspect of their perceived facilitators. Additionally, further research is needed in COPD to understand the barriers and facilitators HCPs perceive towards the use of DHT to determine how these technologies can be most effectively integrated into their workflows and clinical decision-making practices.

## Conclusion

Digital health interventions promise to improve self-management engagement in COPD patients, but many user-adoption issues are still commonly cited in the literature. The findings demonstrated that patients perceive several barriers and facilitators to adopting DHT. Lack of perceived usefulness, illness perception and social context, and digital literacy were all highlighted as barriers to adoption. These findings suggest that future COPD interventions using DHT should consider the use of person-centred design approaches, such as conducting ethnographic user-research in the requirements-gathering phase, to help ease adoption barriers associated with factors of the digital divide. Existing digital self-efficacy, personalised education and community-based support were discussed as facilitators. The findings suggest that future DHT studies in COPD should consider budgeting for added human resources to effectively integrate training and education programmes into their implementation strategies. This paper offers fresh insights regarding the DHT adoption needs of COPD patients, while also highlighting a number of facilitators to help tackle user-adoption issues. These findings contribute a set of key considerations to inform researchers and clinicians about the design of patient-centred study protocols aimed at accounting for the needs and preferences of patients in the development of implementation and adoption strategies for DHT in COPD; however, they should not be relied upon as a substitution for the independent exploration of the adoption needs of other COPD cohorts participating in a digital health intervention study.

## Supplemental Material

Supplemental material for A qualitative study of chronic obstructive pulmonary disease patient perceptions of the barriers and facilitators to adopting digital health technology Click here for additional data file. Supplemental Material for A qualitative study of chronic obstructive pulmonary disease patient perceptions of the barriers and facilitators to adopting digital health technology by Patrick Slevin, Threase Kessie, John Cullen, Marcus W. Butler, Seamas C Donnelly and Brian Caulfield in Digital Health
