# Supplementary material for: A qualitative study of chronic obstructive pulmonary disease patient perceptions of the barriers and facilitators to adopting digital health technology
Source: Digit Health. 2019 Aug 25;5:2055207619871729. doi: 10.1177/2055207619871729 (PMC6710666; doi:10.1177/2055207619871729)
Supplement: Supplemental material for A qualitative study of chronic obstructive pulmonary disease patient perceptions of the barriers and facilitators to adopting digital health technology [file Supplemental_Material.pdf]

Supplementary Data

Sub-Theme	Example Quotation
**Lack of Perceived Usefulness**	It would be different, I mean people are so used to going to the doctor to tell them about the fact they are sick, and if you are going to get them to send their doctor symptoms through the phone, they might be concerned that won't work (Aged 62, Stage 2, Primary)<br><br>Monitoring…I'm not sure it would help all that much because they're up to their eyeballs with work so there is only so much they could do for me and if they have to try to keep watch that I'm doing OK in the home too, I don't think that it's realistic they could do much for me (Aged 69, Stage 2, Primary)<br><br>With the oxygen counter, when I see the levels are low I would either take some oxygen or I would sit down and do the breathing exercises. I use it most days; it's only small and doesn't take long so it's fine. You see, I could use it and then go for a walk out to the gate and it would go back up and if I was in the clinic, sitting there for an hour waiting to see him it could be up at 95, and I'm not sure if there would be any value in the GP knowing that. (Aged 85, Stage 2, Apprenticeship)<br><br>I've had the same GP for 40 years and I think he knows everything about me so I'm not sure what else he would need to know. He's a good doctor and always does the right thing and always gives the prescription I need and always sends me to the hospital when it's needed, you know? (Aged 82, Stage 2, Apprenticeship)
**Digital Literacy**	Em, I think the doctors, or the nurses would have to explain it all to you first. Tell me why they want to do it and what way it will help me. Sure, if it's not explained properly then I might not know what I'm doing, and it could go wrong so that's not good. Then if I don't know what's really going on, I will lose interest quickly. (Aged 65, Stage 3, Secondary)<br><br>Em, you'd have to shown how to use it first and foremost. You can't really use something if you don't get shown first. And like, as I was saying to you earlier, herself helps me so much so she would have to understand too and my daughter as well. Like, that is important cause what happens when I'm sick and I have to send something? Who would do it if they didn't know how and I wasn't fit to show them? What then? (Aged 74, Stage 4, Primary)

	So then, let's say I am given some device to bring home or I have to use something on the phone, say an app, then I will want to fully be clued into how that will work and why it is given to me. (Aged 73, Stage 3, Apprenticeship)<br><br>It would take a fair but of time someone explaining it all to me anyways, and then if it wasn't working right, I wouldn't be able to fix it so someone would have to call here and you kind of don't want to be a hassle, so I don't think them gadgets are for everyone, some will be able for them and others won't so you'd have to be careful who you give them to. (Aged 72, Stage 3, Primary)<br><br>with any technology, been aware of why they have been given it, and then making sure they know what way it will be useful to them, will probably mean that people will get the most worth out of it. Well, if they don't use it right, how will it do them any good? Just like not taking an inhaler right, what good will it be to them? (Aged 57, Stage 4, Secondary)
**Illness Perception and Social Context**	It seems awful to say, cause maybe these things could help, but I feel too, like, I think I am happy to just stay with the way I have always dealt with things. It seems hard to imagine someone giving a phone or a computer and me using it, doesn't it? It would be different if I was in my 60's and had a young family and a job, I would want…it would be more important to try to be as healthy as I could. But I just go from day-today and I get on OK, and that is all I feel I can ask. (Aged 87, Stage 2, Primary)<br><br>It's all part of who you are I think. Definitely some people will be delighted to use their phone or a monitor, but others won't be interested. Well, I'm retired, I have so much time to look into things and do the exercises, I've the time to manage my COPD as best I can. But if I had a few teenage kids in the house, and I was being a busy mum I don't know if I'd be as committed to my COPD as I am. It takes a huge amount of time, to do the exercises, to take the medications, to just be health y in general, and I don't think if I had to be getting them out to school, taking them to hockey or football, that I would be able for it. Same as those who still work or those who have to mind their parents constantly, would it matter to them if they had a monitor? They probably wouldn't be able to keep a diary, let alone a monitor. It all depends on the person and their circumstances, and that's up to the doctor to see if they would get any benefit from these things. (Aged 64, Stage 2, Secondary)<br><br>Like, I feel if these devices are able to collect readings that are useful to the doctors that everyone should be able to avail of them, it just shouldn't be something for the well off. I've a parent who is very sick, with a heart problem also, and I know I could probably chip in to help them buy a monitor, but a lot of elderly people and not just elderly people, but families too,

	that might be able to afford that, and they then are missing out on a treatment, or whatever you want to call it, that might help them a lot. That wouldn't be fair. (Aged 64, Stage 1, Undergraduate)<br><br>Em, it's so depends on the person. There would also be that mindset, am I well enough to do this? I don't want to be taking on new treatments, especially not ones that mean I have to learn to use a monitor if I don't feel up to it. I can't imagine that happening for me anyways. (Aged 61, Stage 2, Undergraduate)<br><br>I don't think I'd be confident no. I mean, I'm just getting by with the COPD and it's not easy thing to keep on top of I'm just sick so much, so it would be another thing to have to do if I was given a monitor. (Aged 73, Stage 2, Primary)

**Sub-Theme**	**Example Quotation**
**Existing Digital Literacy**	It wouldn't matter really. I have had a computer and a laptop for years and I like using them. Much easier to read off them than the phone, not that I can't see the writing on the phone, it's just that it's not such a small screen. (Aged 73, Stage 3, Apprenticeship)<br><br>It'd be grand for me, I'm pretty good with technology, and I can use the oxygen and adjust that no bother, it was easy to learn. So, I think I'd have no problem learning about how to use a monitor, it'd make it fairly comfortable for me, cos I've that confidence. It would also be a case that I'm younger too, so like, it's not that older people like my mother, couldn't use a monitor, it's just that it might take me less time to get the hang of it so a person like me who can pick it up quicker, might stick with it for longer, instead getting completely stressed out by it. (Aged 57, Stage 4, Secondary)<br><br>Em, ah yea, you get more sure of yourself and that yea, I can do it, it's just about learning and once you learn once it's all there for something else. Like, if you learn to drive one car, yea another might be different, but you will pick it up way quicker having driven before! (Aged 65, Stage 2, Secondary)<br><br>Oh, the pendent alarm is fantastic… I'd be long gone without it. I mean, with no one around to see me when things like this happen, this means I'm not alone in an emergency…amazing what they can do these days. It really makes sense for someone as old as me to have one of these, like, it's a security thing isn't it. You get used to working it so I would feel a bit more confident with figuring out another monitor if I had to, even if it was uncomfortable at first, I'd still get on with it cause the alternative is that I just ignore these things and don't get any benefit. (Aged 65, Stage 3, Primary)

**Personalized Education**	You would need to know why it is relevant to your problem, cos it will make it more reasonable for the person, they like, know why it should be used. They mightn't care about it and just leave it altogether otherwise. (Aged 75, Stage 3, Primary)<br><br>I'd be fine with a monitor like, I'd try to learn to use it, but like, I need to be shown what it is for and how to use it. It's hard when you are just learning about the disease, and I don't really know what it's all about, so they give you inhalers and they are grand but it takes a good while to get it right, so you would need to hear this is why you need this, you have these problems and this is where the monitor can help you. (Aged 53, Stage 2, Primary)<br><br>So, you would need to be taught what the reading means, and means for you, cos my oxygen levels may be different to yours during the day, so it would have to geared to me. (Aged 67 Stage 3, Undergraduate)<br><br>Well, education is the keystone of how I look after my COPD. It would be foolish to think that someone with an illness like mine could do anything right for their health if they don't know why they are doing it, like you'd never ask someone to take medication that they don't know how to take or even know what it is for? That'd be dangerous, so even with any technology, been aware of why they have been given it, and then making sure they know what way it will be useful to them, will probably mean that people will get the most worth out of it. (Aged 89, Stage 2, PhD)
**Community Adoption Support**	Like, I'm here on my own, I don't have anyone to tell me that I'm doing this or that wrong, I don't even have someone to cook me a good meal. It's not straight forward at the best of times, I'd really need someone to come to the house and check in and give me pointers for a few weeks, to say, yea, you've got it. (Aged 65, Stage 3, Primary)<br><br>I can see how, as I get older, that I might not be as alert as I am now, I think that just comes with old age territory. I see my Da there, he is nearly 80 and he is a very intelligent man, but over the las few years he has got a good bit slower with even using the remote for the telly. There last year I bought them a new microwave and I had to replace it because they couldn't get the hang of it. Now, it was difficult but my Da even said it to me that if he was younger he would have had the energy to figure it out, but he couldn't get to that point with this one. I'd be wary of that as I get older, some people would definitely

	benefit from one-to-one education to get them more proficient, and then to actually go and see them use the device, that would give them a lot of support to build up their trust in themselves. (Aged 46, Stage 2, Undergraduate)<br><br>It would be great to learn from others who have monitors, like in the clinic, if they could come in and talk to me about using them and I could hear how they are getting on with it? Cos, I reckon there would be a lot of fear there for people that they mightn't be able for it and then seeing other people with COPD, that like, it does help, and they like it, that would help with the process of getting one. (Aged 65, Stage 3, Primary)<br><br>So, with the monitors, it's fine for me to go out and buy them, but it would make more sense to have the GP or the respiratory nurse educate me about them, to watch me use them, because that way they can tell me how to get the best use out of them. I've no background in monitoring, so if I'm learning I mightn't get it or see the point, whereas they might be able to suggest ways of using it that I benefit from, I think everyone would want them involved in process at the start. (Aged 67, Stage 3, Undergraduate)
